# Cranial nerves as pathways for human cerebrospinal fluid efflux: In vivo evidence

**DOI:** 10.1177/0271678X251386232

**Published:** 2025-11-11

**Authors:** Benedicte Falkenberg-Jensen, Are Hugo Pripp, Geir Ringstad, Per Kristian Eide

**Affiliations:** 1 Department of Radiology, Oslo University Hospital—Rikshospitalet, Oslo, Norway; 2 K.G. Jebsen Centre for Brain Fluid Research, University of Oslo, Oslo, Norway; 3 Faculty of Medicine, Institute of Clinical Medicine, University of Oslo, Oslo, Norway; 4 Oslo Centre of Biostatistics and Epidemiology, Research Support Services, Oslo University Hospital, Oslo, Norway; 5 Department of Geriatrics and Internal medicine, Sorlandet Hospital, Arendal, Norway; 6 Department of Neurosurgery, Oslo University Hospital—Rikshospitalet, Oslo, Norway

**Keywords:** CSF efflux routes, brain metabolism, molecular clearance, magnetic resonance imaging, cerebrospinal fluid tracer, trigeminal nerve

## Abstract

In vivo evidence for cerebrospinal fluid (CSF) efflux along cranial nerves in humans is scarce. This study investigated whether the trigeminal, facial, and vestibulocochlear nerves serve as efflux routes for CSF in humans. A magnetic resonance imaging (MRI) contrast agent, used as a CSF tracer, was administered intrathecally at the lumbar level, and consecutive MRI acquisitions measured tracer enrichment along the trigeminal, facial, and vestibulocochlear nerves. The study included 27 patients undergoing evaluation for potential CSF disturbances, but none of whom exhibited evidence of CSF pathology or other neurological diseases. After intrathecal tracer injection, the tracer enriched the prepontine subarachnoid space. Subsequently tracer enrichment was observed within the trigeminal nerve within the subarachnoid space, Meckel’s cave, within the mandibular branch at the foramen ovale and the inferior alveolar nerve in the mandibular bone. The facial nerve was enriched within the subarachnoid space, as well as within the tympanic segment and mastoid segment nearby the stylomastoid foramen. The vestibulocochlear nerve was enriched with tracer within the subarachnoid space. These findings demonstrate that a CSF tracer penetrates the trigeminal, facial, and vestibulocochlear nerves in a peripheral direction, providing evidence that efflux of CSF occurs along cranial nerves in humans.

## Introduction

The pathways through which cerebrospinal fluid (CSF) is cleared from the brain and craniospinal cavity remain a topic of ongoing debate.^1,2^ A variety of efflux routes have been proposed, including clearance through arachnoid granulations into the venous sinuses, lymphatic vessels within the dural meninges, and olfactory nerve rootlets.^1^ Recent experimental studies in rodents have suggested that spinal nerves,^3,4^ vertebral lymphatic vessels,^5,6^ and the trigeminal nerve^7^ may serve as CSF drainage pathways. However, it remains largely unknown whether human cranial and spinal nerves are CSF efflux routes, as in vivo evidence in humans is scarce.

Based on existing knowledge, it is not immediately apparent that human cranial nerves could act as CSF clearance routes.^8,9^ Protective barriers surrounding cranial nerves make them relatively impermeable to substances within CSF. These include the blood–nerve barrier, composed of perineurial cells and tight junctions that limit substance passage; the arachnoid barrier layer, which separates the cranial nerves from CSF as they exit the central nervous system (CNS); and the insulating layers of myelin sheaths and Schwann cells in the peripheral segments of cranial nerves.^9^ Thereby, the efficacy of cranial nerves as CSF efflux routes might be limited. In line with this, a previous human in vivo study presented no evidence of CSF drainage to the nasal mucosa, while tracer along olfactory nerve rootlets was seen.^10^

The present study aimed to investigate whether the trigeminal, facial, and vestibulocochlear nerves could serve as efflux pathways for solutes in the CSF. To test this hypothesis, we administered intrathecally gadobutrol, a hydrophilic magnetic resonance imaging (MRI) contrast agent with a molecular weight of ~604 Da that remains outside the blood–brain barrier, as a CSF tracer. Tracer enrichment was examined at various points along the course of these nerves. The findings support the hypothesis that cranial nerves may play a significant role in CSF clearance.

## Materials and methods

### Approvals and study design

The present study was approved by the relevant authorities: The Regional Committee for Medical and Health Research Ethics (REK), Health Region South-East, Norway (2015/96), the Institutional Review Board at Oslo University Hospital (2015/1868), and the National Medicines Agency (15/04932-7). The study is registered at the Oslo University Hospital Research Registry under ePhorte 2015/1868. The study was conducted in accordance with the ethical standards of the Declaration of Helsinki (1975, revised in 1983) and involved written and oral informed consent from all participants. The study design was prospective and observational.

### Study group and injection procedure

The study included consecutive patients who underwent intrathecal contrast-enhanced MRI as part of their evaluation for suspected cerebrospinal fluid (CSF) disorders at the Department of Neurosurgery, Oslo University Hospital—Rikshospitalet, Norway. The clinical indication for MRI in these patients was assessment of tentative CSF flow alterations; they were symptomatic individuals with cysts (arachnoid or pineal) or with headache in association tentative idiopathic intracranial hypertension or hypotension. After thorough diagnostic work-up in the presently included subjects, no CSF abnormalities or neurological diseases were identified. They were therefore considered to be close to healthy. However, healthy control subjects were not included, as intrathecal gadobutrol is currently administered off-label at our institution based on clinical indications. Therefore, the intrathecal contrast-enhanced MRI is performed in research setting after written and oral informed consent.

The MRI contrast agent gadobutrol (Gadovist; Bayer Pharma AG, Berlin, Germany) was injected intrathecally at the lumbar level, in a dose of 0.5 mmol (0.5 ml of 1.0 mmol/ml gadobutrol), acting as CSF tracer. The injection was performed by an interventional neuroradiologist.

### MRI protocol

MRI data were acquired using a 3 T Philips Ingenia MRI scanner (Philips Medical Systems, Best, The Netherlands). Standardized T1-weighted MRI scans were performed before and at multiple time points following the intrathecal gadobutrol injection: ⩽1, 6 h (day 1), 24 h (day 2), and 48 h (day 3). Gadobutrol enhances T1 signal due to increased T1 relaxation of water, allowing semi-quantitative measurement of tracer levels.

Sagittal 3D T1-weighted volume scans were acquired at each time point with the following imaging parameters: repetition time = shortest (typically 5.1 ms), echo time = shortest (typically 2.3 ms), flip angle = 8°, field of view = 256 × 256 mm, and matrix = 256 × 256 pixels (reconstructed to 512 × 512). A total of 184 overlapping slices (1 mm thickness) were sampled, and the images were automatically reconstructed to 368 slices with 0.5 mm thickness. The duration of each scan was 6 min and 29 s. To ensure consistency and reproducibility in slice placement and orientation, an automated anatomy recognition protocol (SmartExam™; Philips Medical Systems, Best, The Netherlands) based on landmark detection was used for every time point.

### Image analysis

Changes in T1 signal were evaluated within regions of interest (ROIs) along the different cranial nerves, which were placed inside the nerve in well distance to the outer nerve border to avoid partial volume effects. To emphasize differences in subtle signal intensities, we applied a color palette in addition to shades of gray. The color palette feature in Sectra PACS (Sectra AB, Linköping, Sweden) modifies the way grayscale MRI images are displayed by mapping different intensity levels to a specific color gradient. The underlying pixel values remain unchanged; only the visual representation is altered. This approach enhances contrast perception and improves visualization of subtle details that might be difficult to distinguish in a standard grayscale display. All measurements were done by an experienced ear–nose–throat (ENT) radiologist with 19 years of experience.

**Figure 1.**
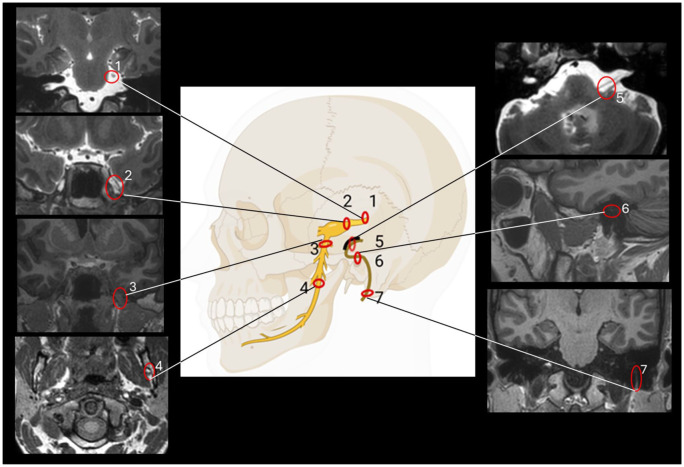
Overview of locations for assessing tracer enrichment along the trigeminal nerve (left) and along the facial nerve (right). For the vestibulocochlear nerve assessment was done within the subarachnoid space (corresponding to location 5).

**Figure 2.**
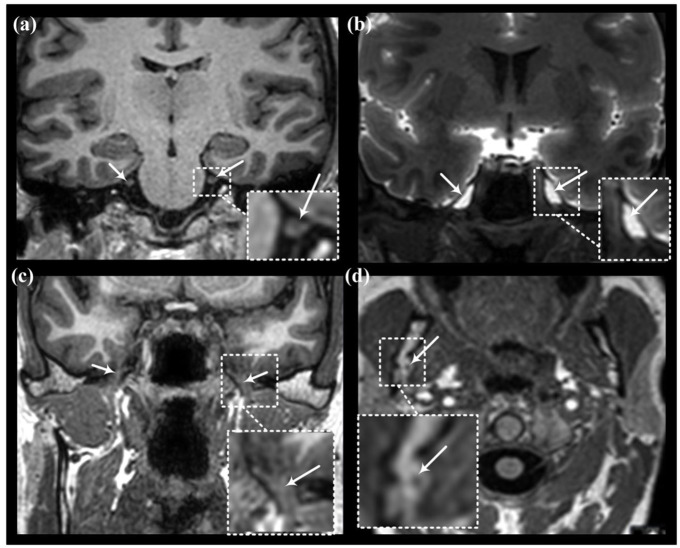
Slice sections and measurement areas along the trigeminal nerve utilizing T1-weighed MRI. Images demonstrate slice sections of the trigeminal nerve and branches at (a) prepontine subarachnoid space in coronal plane, (b) Meckel’s cave in coronal plane, (c) foramen ovale in coronal plane, and (d) mandibular bone in axial plane. White arrows point at the nerve segments; for Meckel’s cave the region of interest was within the cave itself. MRI: magnetic resonace imaging.

**Figure 3.**
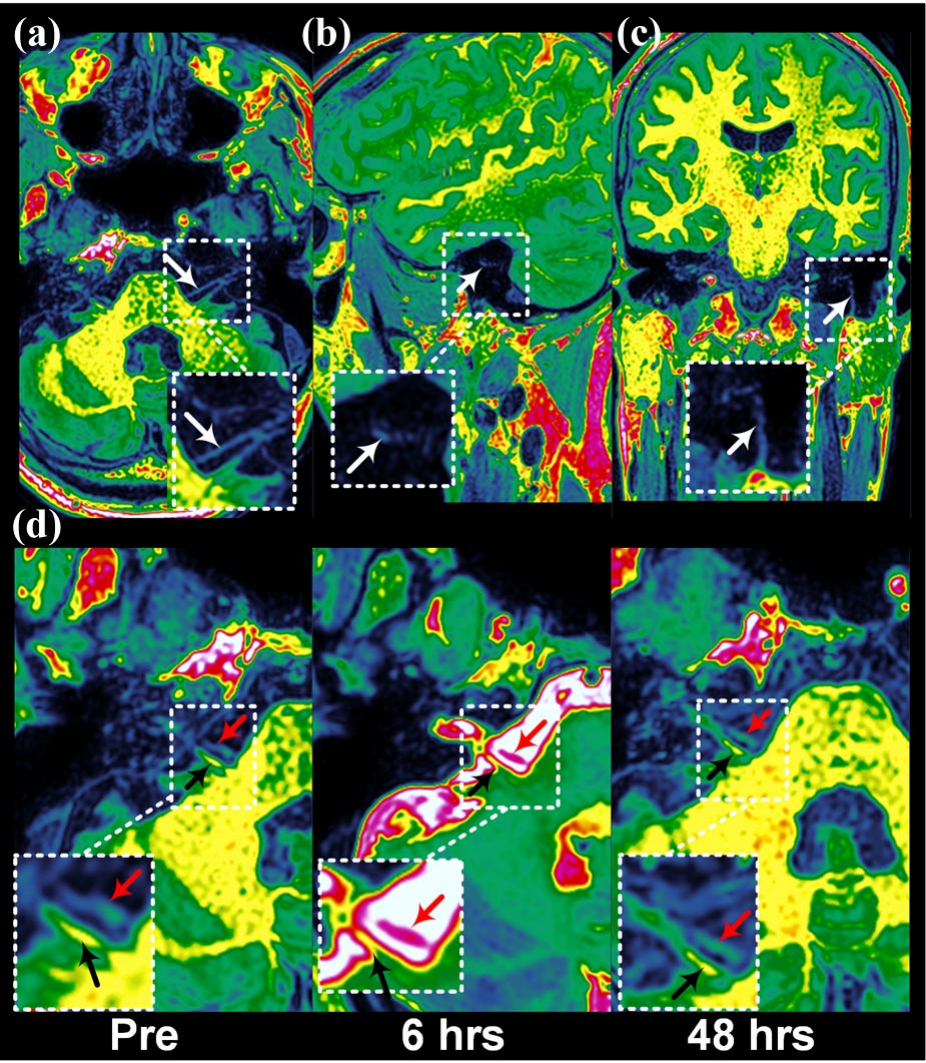
Slice sections and measurement areas along the facial and vestibulocochelar nerves utilizing T1-weighed MRI where the grayscale images are displayed with a color palette (“French,” SECTRA Medical) where the amount of signal in each voxel determines the color, based on the intensity in the grayscale image. The color is for illustration and better visualization and does not represent a change of baseline signal intensity. Images in upper row demonstrate slice sections at different segments of the facial nerve (white arrow) at (a) prepontine subarachnoid space in axial plane, (b) tympanic segment in sagittal plane, (c) mastoid segment at stylomastoid foramen in coronal plane. Axial images at lower row show the (d) vestibulochochlear nerve (black arrow) and facial nerve (red arrow) before (pre) and 6 and 48 h after intrathecal tracer. MRI: magnetic resonace imaging.

Measurements were made bilaterally along the trigeminal nerve, the facial nerve, and the vestibulocochlear nerve before contrast injection, and 6, 24, and 48 h after injection. An overview of the locations for assessing tracer enrichment along the nerves is shown in Figure 1. The trigeminal nerve was measured in its cisternal portion, tracer enrichment within the Meckel’s cave was assessed, and tracer enrichment was assessed within the mandibular branch at its exit from the oval foramina, and within the nerve branch at its entrance to the mandibular canal (Figure 2(a)–(d) and Supplementary Figures 1 and 2). Enrichment along the facial nerve was measured in three locations, that is, in the prepontine subarachnoid space, within the tympanic segment and the mastoid segment (Figure 3(a)–(c) and Supplementary Figure 3). Enrichment of the vestibulocochlear nerve was measured within the prepontine subarachnoid space (Figure 3(d)). Examples of placement of ROIs are given in Supplementary Figure 4.

To correct for changes in image grayscale between MRI scans, the T1 signal within each ROI at each time point was divided by the T1 signal of a reference ROI at the respective time point. The reference ROI was placed in the posterior part of the orbit, as described previously.^11^ The resulting ratio represents the normalized T1 signal, allowing correction for baseline changes in image grayscale, such as those introduced by automatic image scaling. Accordingly, tracer enrichment was semi-quantified as percentage increase in normalized T1 signal, identical to the approach we have consistently used in other studies.^12,13^

### Statistical analyses

Continuous data are presented as means (standard deviations) and categorical data as numbers of observations. The Pearson’s correlation test was used to assess correlations between variables. Repeated measurements were analyzed with linear mixed models with maximum likelihood estimation and a subject-specific random intercept; results were visually presented as margin plots displaying means and 95% confidence intervals. We used a mixed linear model to evaluate differences between the repeated measurements in a single overall statistical model. This approach was deemed statistically more robust than using a set of basic statistical tests. Statistical significance was accepted at the 0.05 level (two-tailed). All the statistical analyses were performed using Stata version 18 (StataCorp LLC, College Station, TX, USA) or RStudio version 2024.09.1 (Posit PBC, Boston, MA, USA) with R version 4.4.0 (R Foundation for Statistical Computing, Vienna, Austria).

## Results

### Patient cohort

The study included 27 consecutive individuals undergoing evaluation for potential CSF disturbances, but in whom no evidence of CSF failure or other neurological diseases was found. Details of the patient cohort are presented in Supplementary Table 1.

### CSF tracer enrichment along the trigeminal nerve

**Figure 4.**
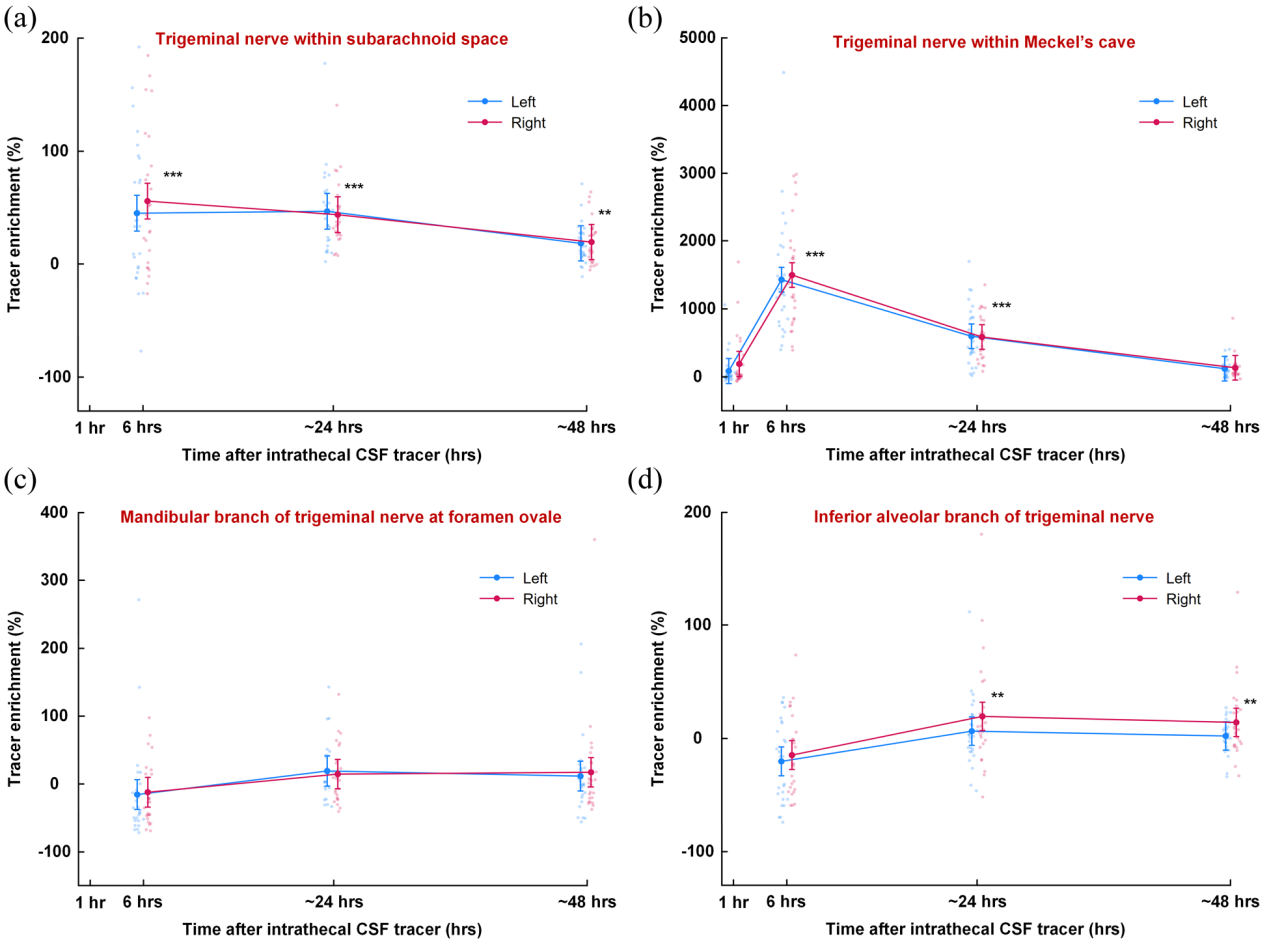
Percentage change in tracer enrichment compared to pre-injection levels along the trigeminal nerve. The percentage change in tracer enrichment at various time points after intrathecal tracer injection is shown for the left (blue) and right (red) trigeminal nerves at (a) the prepontine subarachnoid space, (b) CSF within Meckel’s cave, (c) the foramen ovale, and (d) inferior alveolar branch at the mandibular bone. **p < 0.01, ***p < 0.001 from test of tracer enrichment of both sides combined. Trend plots are presented as means ± 95% CIs, derived from linear mixed models, along with individual values. 95% CIs: 95% confidence intervals.

Intrathecal administration of the tracer (gadobutrol) resulted in enrichment within the CSF of the prepontine cistern (Supplementary Figure 5). The time from intrathecal injection to appearance of tracer in cisterna magna (spinal transit time) was on average 15.8 min (Supplementary Table 1). Tracer enrichment was observed along various segments of the trigeminal nerve, including the nerve within the subarachnoid space (Figure 4(a)), within the Meckel’s cave where we measured CSF itself (Figure 4(b)), and within the mandibular nerve at the foramen ovale (Figure 4(c)), and within the inferior alveolar nerve within the mandibular bone (Figure 4(d)). Comparing tracer enrichment after 6 h with enrichment after 24 and 48 h demonstrated a significant signal increase within the trigeminal nerve in the subarachnoid space (24 h: *p* = 0.504; 48 h: *p* < 0.001; Figure 4(a)), in CSF of Meckel’s cave (24 h: *p* < 0.001; 48 h: *p* < 0.001; Figure 4(b)), and within the mandibular branch at the foramen ovale (24 h: *p* = 0.001; 48 h: *p* = 0.001; Figure 4(c)), and within the inferior alveolar nerve in the mandibular bone (24 h: *p* < 0.001; 48 h: *p* < 0.001; Figure 4(d)). Enrichment was strongest at the proximal part of the nerve but remained significant in the peripheral sections.

**Figure 5.**
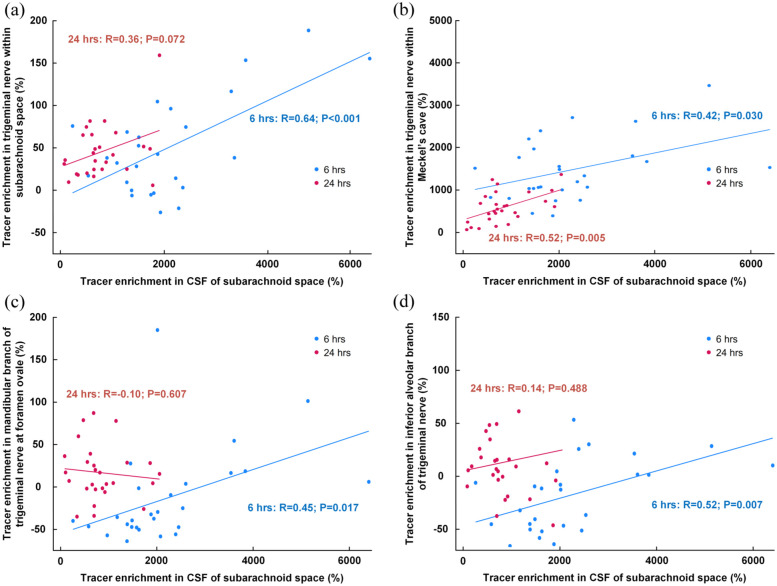
Association between tracer enrichment in subarachnoid space and along the trigeminal nerve. The correlation between percentage change in tracer enrichment at 6 h (blue dots and line) and 24 h (red dots and lines) within the CSF of the subarachnoid space and the tracer enrichment in (a) the trigeminal nerve within the subarachnoid space, (b) trigeminal nerve within the Meckel’s cave, (c) in the mandibular branch of the trigeminal at the foramen ovale, and (d) inferior alveolar branch of the trigeminal nerve. For scatter plots the fit lines and Pearson correlation coefficients with significance levels are shown.

There was a strong positive correlation between tracer enrichment in CSF of subarachnoid space and enrichment along the trigeminal nerve, most significant at 6 h than at 24 h, including within the trigeminal nerve of the subarachnoid space (Figure 5(a)), CSF within Meckel’s cave (Figure 5(b)), within the mandibular branch at foramen ovale (Figure 5(c)), and within the inferior alveolar nerve (Figure 5(d)).

There were no differences in tracer enrichment between the left and right nerves (Supplementary Figure 6), although inter-individual variability was evident.

### CSF tracer enrichment along the facial and vestibulocochlear nerves

**Figure 6.**
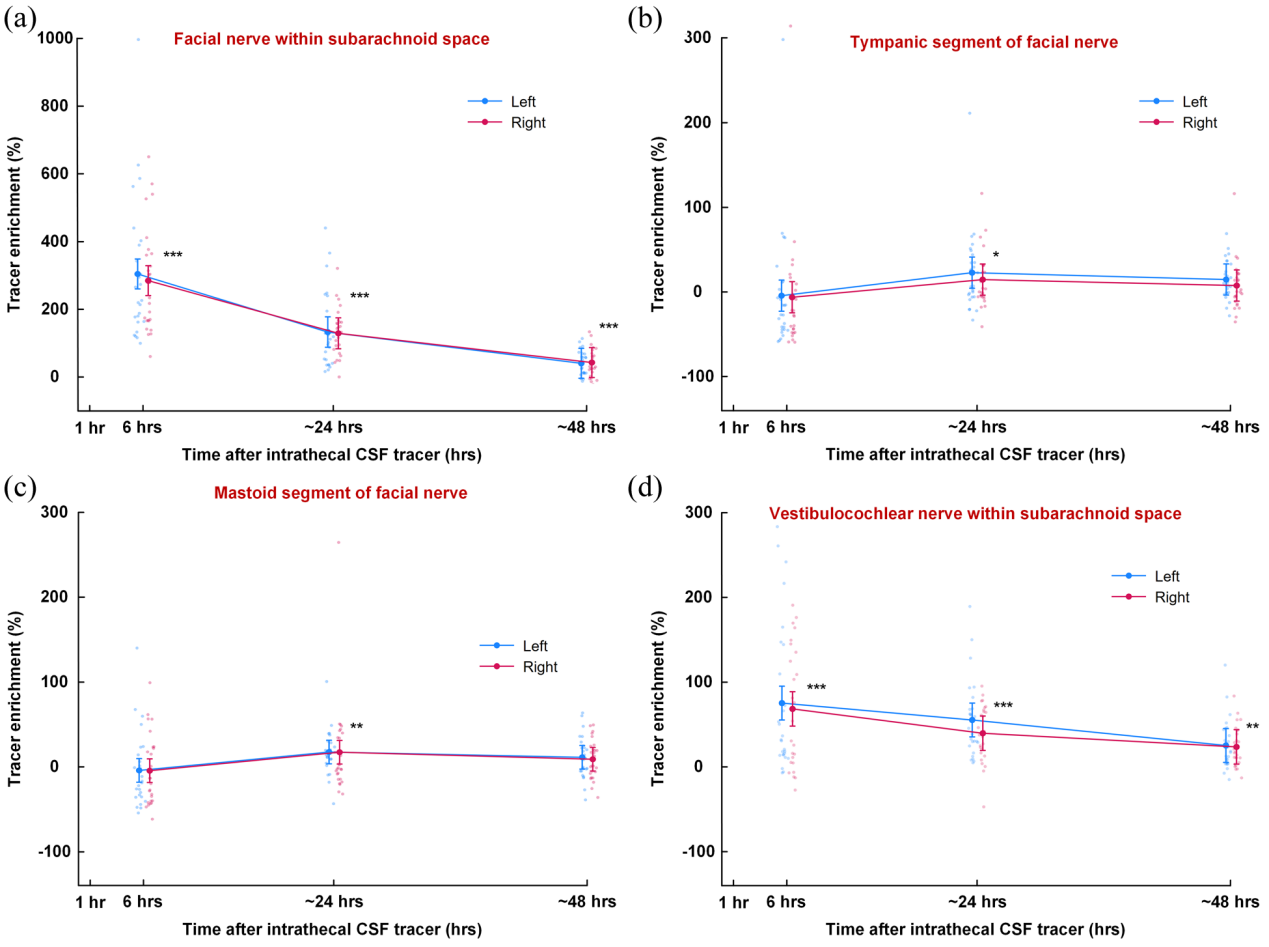
Percentage change in tracer enrichment compared to pre-injection levels along the facial and vestibulocochlear nerves. The percentage change in tracer enrichment at various time points after intrathecal tracer injection is shown for the left and right facial nerves at (a) the prepontine subarachnoid space, (b) the tympanic segment, (c) the mastoid segment at the stylomastoid foramen, and (d) the vestibulocochlear nerve with the prepontine subarachnoid space. *p < 0.05, **p < 0.01, ***p < 0.001 from test of tracer enrichment of both sides combined. Trend plots are presented as means ± 95% CIs, derived from linear mixed models, along with individual values. 95% CIs: 95% confidence intervals.

Tracer enrichment along the facial nerve was observed within the facial nerve in the CSF of the subarachnoid space (Figure 6(a)), and within the tympanic (Figure 6(b)) mastoid segments of the nerve (Figure 6(c)). As compared with tracer enrichment after 6 h, enrichment was significantly increased after 24 and 48 h within the facial nerve segment in subarachnoid space (24 h: *p* < 0.001; 48 h: *p* < 0.001; Figure 6(a)), the tympanic segment (24 h: *p* = 0.002; 48 h: *p* = 0.036; Figure 6(b)), and mastoid segment (24 h: *p* = 0.001; 48 h: *p* = 0.002; Figure 6(c)). Strong enrichment also was found within the vestibulocochlear nerve within the subarachnoid space (Figure 6(d)).

**Figure 7.**
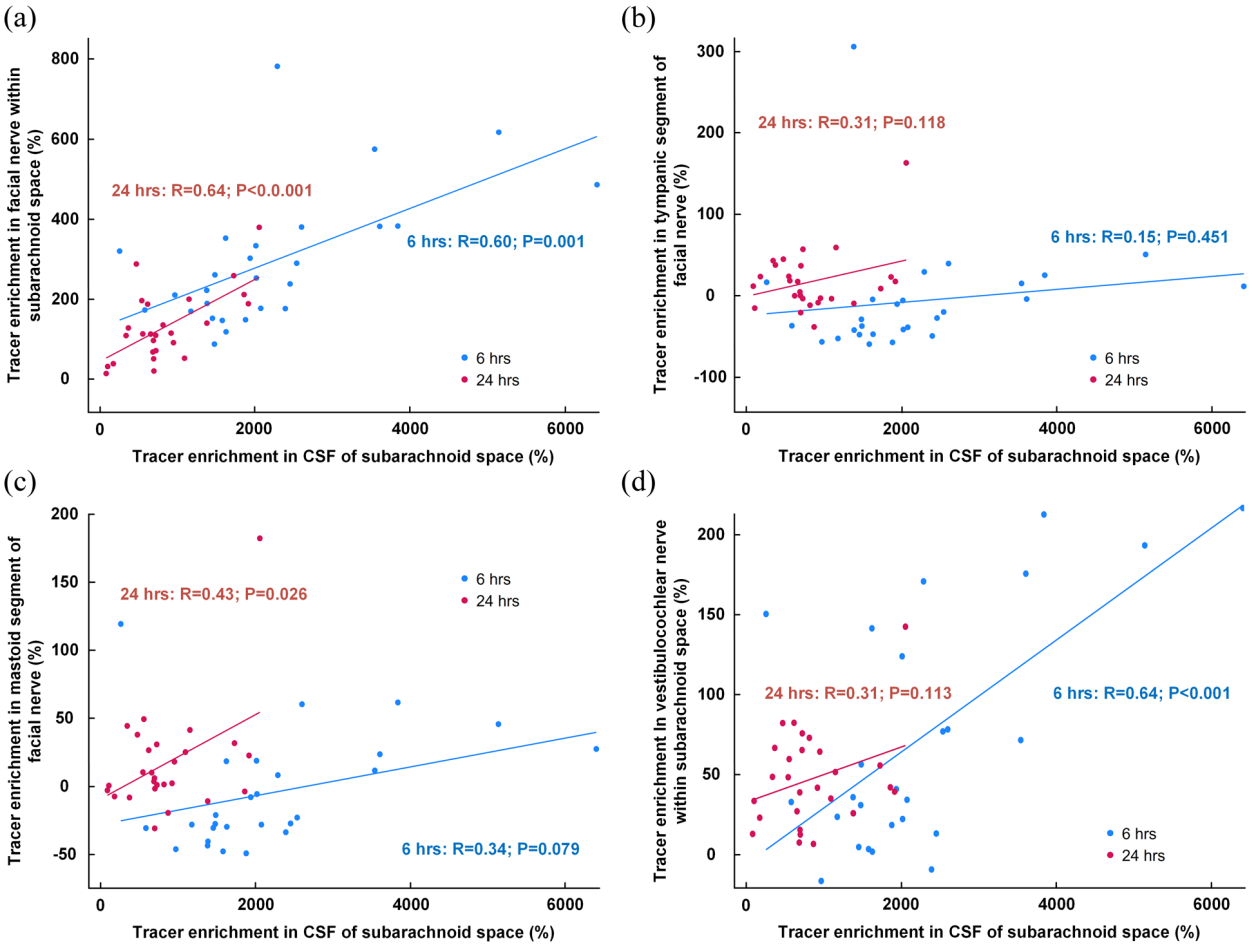
Association between tracer enrichment in subarachnoid space and the facial and vestibulocochlear nerves. The correlation between percentage change in tracer enrichment at 6 h (blue dots and line) and 24 h (red dots and lines) within the CSF of the subarachnoid space and the tracer enrichment in (a) the facial nerve within the subarachnoid space, (b) tympanic segment of the facial nerve, (c) the mastoid segment at the stylomastoid foramen, and (d) the vestibulocochlear nerve within the CSF of the subarachnoid space. For scatter plots the fit lines and Pearson correlation coefficients with significance levels are shown.

We found a positive correlation between tracer enrichment in CSF of subarachnoid space and enrichment in the facial nerve at 6 and 24 h (Figure 7(a)), no correlation within the tympanic segment (Figure 7(b)), but a positive correlation between tracer enrichment in CSF and the mastoid segment at 24 h (Figure 7(c)). Furthermore, there was a highly significant positive correlation between tracer enrichment within CSF and the vestibulocochlear nerve of the subarachnoid space (Figure 7(d)).

No differences in tracer enrichment were observed between the left and right sides of the facial nerve (Supplementary Figure 7(a)–(c)) or the vestibulocochlear nerve (Supplementary Figure 7(d)), but inter-individual variability was again evident.

## Discussion

The key finding of this study is that a CSF tracer within the prepontine subarachnoid space demonstrates significant enrichment along the trigeminal, facial, and vestibulocochlear nerves in a peripheral direction. The degree of enrichment correlates strongly with tracer enrichment within CSF of prepontine subarachnoid space. This observation provides evidence that cranial nerves may act as efflux pathways for CSF in humans, highlighting a previously underappreciated mechanism of CSF clearance.

The presently used CSF tracer (gadobutrol) of 604 Da molecular size has hydrodynamic diameter <2.6 nm, comparing well with the 1.9 nm gold particles recently reported to pass from CSF along spinal nerves to the peripheral part of the body.^3^

The present findings differ from previous human studies, as in vivo evidence for CSF efflux along cranial nerves to the periphery has not been demonstrated before. Varatharaj et al.^14^ reported a case of multiple sclerosis in which intravenous gadolinium administration resulted in tracer enrichment within the trigeminal nerve. The authors speculated that this might indicate CSF clearance along the trigeminal nerve. However, following intravenous contrast administration, the exact transport mechanism remains unclear, as the tracer may have reached the nervous tissue via alternative routes. Our group previously showed no enrichment in the nasal mucosa near the olfactory nerve.^10^ Due to the small size of the olfactory rootlets within the subarachnoid space, enrichment within these are difficult to visualize. On the other hand, others have provided evidence of CSF efflux along the olfactory nerve.^15^ However, the olfactory nerve seems not to have any significant role as a CSF efflux pathway in humans, tentatively due to the small size of the human olfactory nerve rootlets. We also found enrichment of an intrathecal tracer along the course of the optic nerve, but without any evidence of enrichment beyond the nerve itself—neither within the ocular bulb nor along the sclera.^16^ Therefore, the present observations represent the first in vivo human evidence for efflux of solutes along the peripheral course of cranial nerves.

A previous study in rats has shown that glymphatic transport of an intrathecal tracer (Magnevist) depends on body posture.^17^ This raises the question of whether the clearance capacity of individual human cranial nerves might be influenced by their degree of vertical orientation, given the upright posture of bipedal humans. Could gravity make the more vertically oriented cranial nerves (e.g. cranial nerves V, VII, IX, and X) more effective as efflux pathways than the more horizontally oriented cranial nerves (e.g. cranial nerves I, II, III, IV, and VIII)? While this is clearly speculative, it is a hypothesis that could be explored in future studies.

The role of spinal nerves as a CSF efflux route is presently unclear. A recent cadaveric study provided experimental evidence that spinal nerves may serves as a CSF efflux route.^18^ In line with this, estimating CSF clearance of a CSF tracer administered intrathecal suggest that clearance along spinal nerves within the thecal compartment plays a significant role in CSF clearance.^19,20^ Hypothetically, there is clearance of substances from CSF of the spinal subarachnoid space, via spinal nerves to lymphatic vessels in the nerve’s dura/ nerve sheath. This pathway may work in concert with clearance directly to lymphatic vessels within dura of the thecal sac.^4,5^

In contrast to the lacking human evidence for CSF efflux along cranial and spinal nerves, a substantial body of experimental research, particularly in rodents, has explored potential CSF efflux pathways along nerves. As recently discussed by Proulx,^1^ most evidence for CSF clearance along cranial nerves focuses on the olfactory and optic nerves. Rodent studies suggest that the olfactory nerve may be a major efflux route. However, species differences should be considered: compared to humans, the rodent olfactory nerve is larger and serves vital functions, which may influence its role in CSF clearance. Another example of species differences is the much stronger tracer enrichment in pons of rodents^21^ than in humans.^12^ Notably, ex vivo studies in rats have indicated the possibility of solute drainage from the subarachnoid space CSF via spinal nerves. In these studies, solutes such as Evans blue-albumin, visualized using fluorescence microscopy, and lanthanum, examined by electron microscopy, were injected into the subarachnoid space of the cisterna magna.^22^ After 24 h, these tracers were detected in the extracellular spaces of ventral and dorsal nerve roots as well as the distal portions of spinal nerves. However, a caveat of such tracer studies is the potential artifact introduced by the volume and pressure changes resulting from tracer injection. These factors may influence the observed patterns of tracer distribution and should be considered when interpreting the results. With regard to the trigeminal, facial, and vestibulocochlear nerves as CSF efflux routes for CSF, the experimental research is conflicting for the trigeminal nerve, but the experimental evidence is more consistent regarding direct communication between CSF and perilymph (for review, see Proulx^1^). Notably, after intrathecal injection of a MRI contrast agent (Magnevist) to rats, enrichment of the tracer was noted at the cranial exit sites of the vestibular and vagal nerves as well as within the cohlea,^17^ suggesting CSF efflux along these nerves.

A key question is how the CSF tracer in this study travels along cranial (and spinal) nerves, given the barrier functions of the nerve sheaths (peri-, epi-, and endoneural sheaths). The nuclei of the cranial nerves we studied are not visualized at MRI and can therefore not be assessed directly with regards to tracer enhancement. However, it is known from previous studies that the pons, harboring these nuclei, has very limited glymphatic enrichment compared to the cortex.^12^ The presently reported correlation between tracer concentration in the CSF and its enrichment in the trigeminal, facial, and vestibulocochlear nerves supports the hypothesis of direct diffusion into the nerve, driven by a concentration gradient from higher to lower levels. This challenges the notion that nerve sheaths are largely impermeable to substances within CSF. Since the CSF tracer used in this study does not cross the blood–brain barrier or enter cells, its passage appears to be entirely extravascular, traveling along nerve sheaths in a peripheral direction. According to this hypothesis, glymphatic clearance occurs into the CSF of the subarachnoid space and from there via the cranial nerves to dural lymphatics.

Another critical question is to which locations the solutes exit from cranial and spinal nerves. In previous work, we demonstrated that a CSF tracer in the subarachnoid space enriches the parasagittal dura (PSD),^23^ which houses lymphatic vessels.^24,25^ Additionally, tracer enrichment was observed in cranial skull bone marrow^26^ and extracranial lymph nodes.^27^ Experimental studies have shown that lymphatic vessels within the dura mater can drain solutes from CSF into the lymphatic system.^24,25^ From this, we hypothesize that tracer efflux from nerves occurs via lymphatic vessels surrounding the nerve sheaths, with subsequent transport through these lymphatic structures and via deep cervical lymph nodes to the systemic circulation.

The direct passage of solutes from the CSF into cranial nerves may have significant implications for the pathophysiology and treatment of various diseases. The trigeminal nerve, primarily a sensory nerve responsible for transmitting sensory input from the facial region and head, is implicated in numerous pain disorders, including trigeminal neuralgia, trigeminal neuropathic pain, and migraines.^28^ It is plausible to speculate that substances within the CSF could influence the trigeminal nerve by either sensitizing or desensitizing it. For instance, neuroinflammation has been proposed as a contributing factor in trigeminal neuralgia.^29^ Allergic inflammation has also been shown to sensitize the trigeminal nerve, potentially through the overexpression of 5-hydroxytryptophan (5-HT) 3 receptors.^30^ Moreover, studies have identified elevated levels of inflammatory mediators in the CSF of patients with trigeminal neuralgia,^31,32^ which may modify nerve function in these individuals. Additionally, recent findings indicate that migraine-associated cortical spreading depressions can activate the trigeminal nerve,^7^ further underscoring the complex interplay between CSF solutes, inflammation, and trigeminal nerve function. Another perspective is that neuroinflammation could be associated with altered CSF clearance capacity. In this regard it is noteworthy that Cheng et al.^33^ reported an increased risk of dementia in patients with trigeminal neuralgia. An interesting speculation is whether trigeminal neuralgia could be accompanied with impaired CSF efflux of waste products. Further studies are required to answer this. Nevertheless, the new insights highlight the potential for targeting CSF-mediated mechanisms in the treatment of trigeminal nerve-related disorders.

It is important to note that there was considerable inter-individual variability in the degree to which the CSF tracer enriched the trigeminal, facial, and vestibulocochlear nerves (Supplementary Figures 6 and 7). CSF clearance of tracer to blood also shows great inter-individual variability.^20^ This variability could potentially affect the efficacy of intrathecal drug therapies, calling for individual assessment of CSF clearance capacity prior to intrathecal drug delivery.

We also note that tracer enrichment was stronger in the facial than the trigeminal and vestibulocochlear nerves within the subarachnoid space (Figures 4 and 6). We have no explanation to this finding, but the observation could possibly be related to the smaller dimension of the facial nerve.

We acknowledge certain limitations in this study. The assessment of tracer enrichment was semi-quantitative, based on the percentage increase in normalized T1 signal. A fully quantitative analysis would require T1 mapping, but current resolution limitations prevent its use for this purpose. Additionally, the precise placement of regions of interest (ROIs) could introduce partial volume effects, potentially influencing the measured T1 signal. This possible limitation was, however, taken into account when placing ROIs. Moreover, the close agreement in observations between the left and right sides suggests that this limitation had minimal impact on the overall findings.

## Conclusions

In conclusion, the present data provide in vivo evidence for the efflux of substances from the CSF to the periphery via the trigeminal, facial, and vestibulocochlear nerves. Given the relatively large size and anatomical significance of these nerves, they could play a significant role in CSF clearance of humans. This finding not only improves our understanding of the mechanisms involved in CSF circulation but also opens promising therapeutic possibilities. Specifically, these observations highlight the potential for treating conditions related to these cranial nerves through targeted interventions via the CSF. This could lead to novel approaches for managing diseases such as trigeminal neuralgia, migraine, and vestibulocochlear disorders, offering a more direct and potentially effective treatment pathway. Further research is needed to fully explore these mechanisms and their implications for clinical applications.

## Supplemental Material

sj-pdf-1-jcb-10.1177_0271678X251386232 – Supplemental material for Cranial nerves as pathways for human cerebrospinal fluid efflux: In vivo evidence Supplemental material, sj-pdf-1-jcb-10.1177_0271678X251386232 for Cranial nerves as pathways for human cerebrospinal fluid efflux: In vivo evidence by Benedicte Falkenberg-Jensen, Are Hugo Pripp, Geir Ringstad and Per Kristian Eide in Journal of Cerebral Blood Flow & Metabolism
