# Supplementary material for: Cranial nerves as pathways for human cerebrospinal fluid efflux: In vivo evidence
Source: J Cereb Blood Flow Metab. 2025 Nov 11:0271678X251386232. Online ahead of print. doi: 10.1177/0271678X251386232 (PMC12611735; doi:10.1177/0271678X251386232)
Supplement: sj-pdf-1-jcb-10.1177_0271678X251386232 – Supplemental material for Cranial nerves as pathways for human cerebrospinal fluid efflux: In vivo evidence [file sj-pdf-1-jcb-10.1177_0271678X251386232.pdf]

Supplementary Material

Cranial Nerves as Pathways for Human Cerebrospinal Fluid Efflux: In Vivo Evidence

Benedicte Falkenberg-Jensen[1,2,3], Are Hugo Pripp[2,4], Geir Ringstad[1,2,3,5], Per Kristian Eide[1,2,3,6*]

[1]*Department of Radiology, Oslo University Hospital- Rikshospitalet, Oslo, Norway.*
[2]*K.G. Jebsen Centre for Brain Fluid Research, University of Oslo, Oslo, Norway.*
[3]*Institute of Clinical Medicine, Faculty of Medicine, University of Oslo, Oslo, Norway.*
[4]*Oslo Centre of Biostatistics and Epidemiology, Research Support Services, Oslo University Hospital, Oslo, Norway.*
[5]*Department of Geriatrics and Internal medicine, Sorlandet Hospital, Arendal, Norway.*
[6]*Department of Neurosurgery, Oslo University Hospital – Rikshospitalet, Oslo,*

***Corresponding author:**
Professor Per Kristian Eide, MD PhD
Department of Neurosurgery
Oslo University Hospital - Rikshospitalet
Pb 4950 Nydalen,
Phone: +47 91649419
Fax: +47-23074310
N-0424 Oslo, Norway
p.k.eide@medisin.uio.no

Supplementary Table 1. Demographic information about the patient cohort

N	27
Sex (F/M)	21/6
Age (years)	39.0 ± 11.7
BMI (kg/m^2)	27.7 ± 5.1
Spinal transit time (min)	15.8 ± 29.8

Supplementary Figure 1

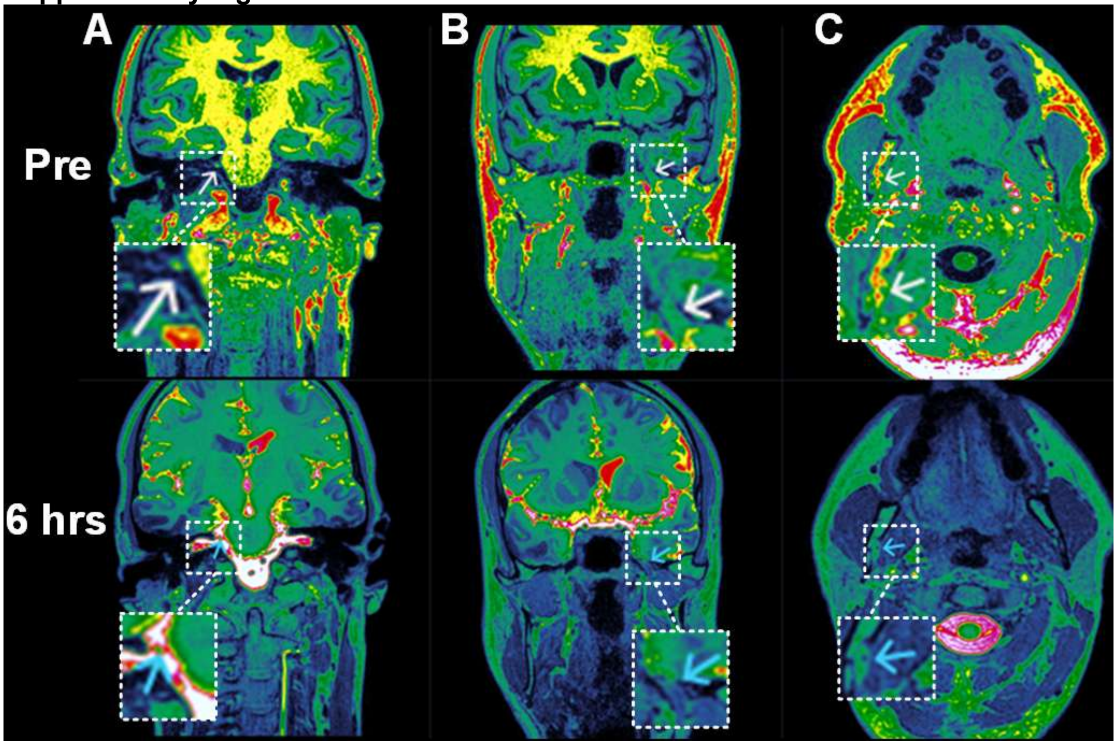

Segments along the trigeminal nerve. For identification of anatomical structures, we used T1-weighed magnetic resonance imaging (MRI), where the gray-scale images are displayed with a color palette ("French", SECTRA Medical) and the amount of signal in each voxel determines the color, based on the intensity in the greyscale image. The color is for illustration and better visalisation, and does not represent a change of baseline signal intensity. The upper row shows images before intrathecal tracer (white arrows point at trigeminal nerve) and the lower row (blue arrows point at trigeminal nerve) shows images 6 hours after intrathecal tracer. The column to left shows the trigeminal nerve within the prepontine subarachnoid space in coronal plane (A), middle column the third branch of the trigeminal nerve at the oval foramen in coronal plane (B) and the column to right shows the mandibular branch within the mandibular bone in axial plane (C). The color palette feature modifies the way grayscale MRI images are displayed by mapping different intensity levels to a specific color gradient. This can enhance contrast perception and improve visualization of subtle details that might be difficult to distinguish in a standard greyscale display.

Supplementary Figure 2

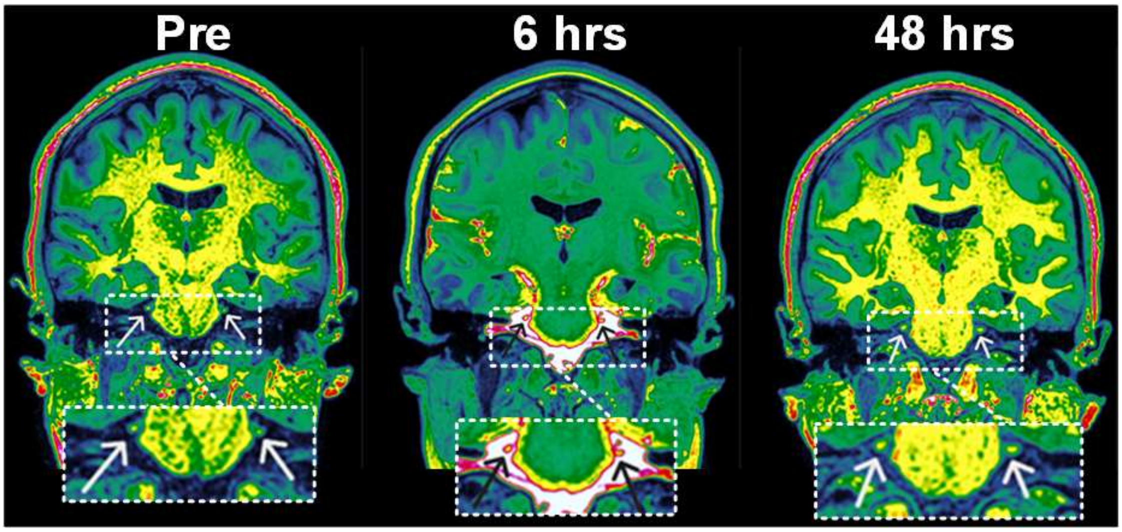

Trigeminal nerve within the prepontine cistern at different times. Coronal images show the trigeminal nerve before intrathecal contrast (Pre) and 6 and 48 hours after intrathecal contrast. Arrows point at the nerve. The images show T1-weighed magnetic resonance imaging (MRI), where the gray-scale images are displayed with a color palette (“French”, SECTRA Medical) and the amount of signal in each voxel determines the color, based on the intensity in the greyscale image. The color is for illustration and better visualization and does not represent a change of baseline signal intensity.

Supplementary Figure 3

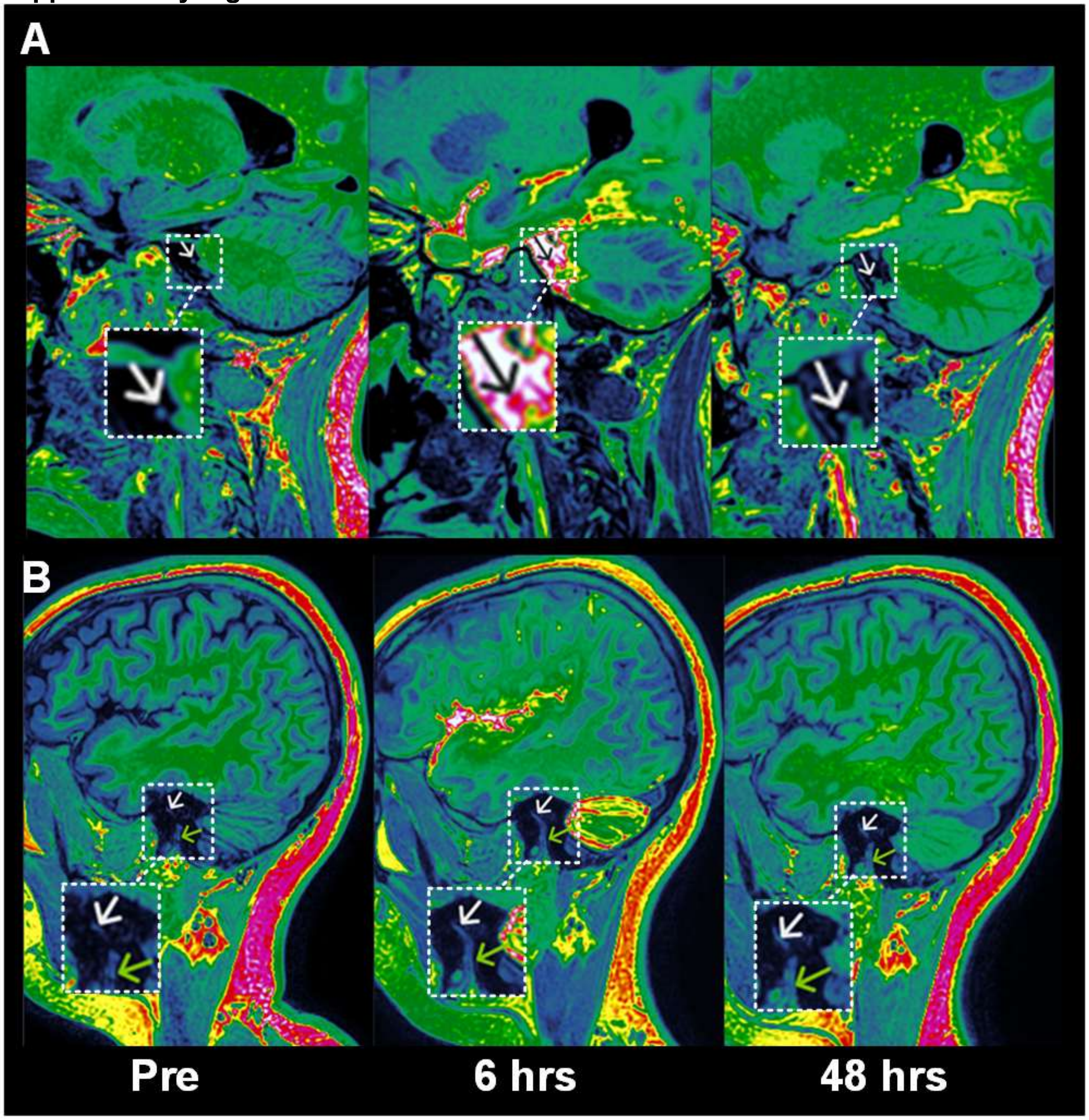

Different segments of the facial nerve at various time points. The column to the left display sagittal images before intrathecal contrast, and sagittal images are shown 6 hours (middle column, sagittal plane) and 48 hours (right column, sagittal plane) after intrathecal tracer. The upper row shows the facial nerve within the prepontine subarachnoid space, while the lower row shows the tympanic segment (white arrows) and the stylomastoid segment (green arrows). The images show T1-weighed magnetic resonance imaging (MRI), where the gray-scale images are displayed with a color palette ("French", SECTRA Medical) and the amount of signal in each voxel determines the color, based on the intensity in the greyscale image. The color is for illustration and better visualization and does not represent a change of baseline signal intensity.

Supplementary Figure 4

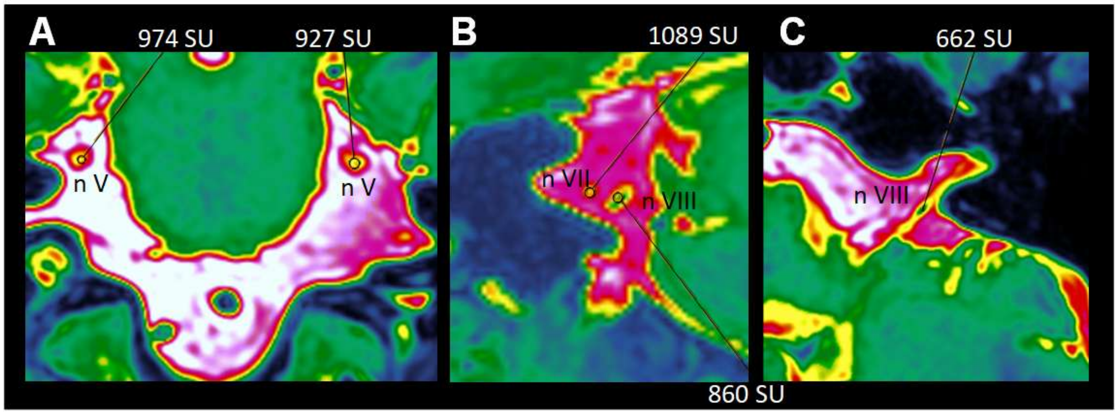

Examples of placement of regions of interest (ROIs). Each ROI was placed well inside the nerve with good margin to outer nerve border to avoid partial volume effects, here illustrated by the trigeminal nerves in coronal plane (A), the facial and vestibulocochlear nerves in sagittal plane (B), and vestibulocochlear nerve in axial plane (C). The signal units are given for the individual ROIs. The images show T1-weighed magnetic resonance imaging (MRI), where the gray-scale images are displayed with a color palette ("French", SECTRA Medical) and the amount of signal in each voxel determines the color, based on the intensity in the greyscale image. The color is for illustration and better visualization and does not represent a change of baseline signal intensity.

Supplementary Figure 5

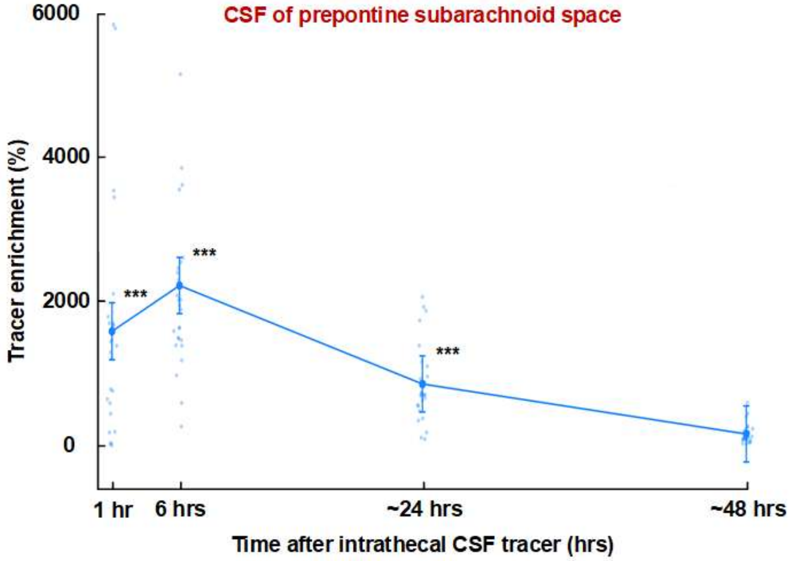

Time evolution of tracer enrichment in the subarachnoid space (SAS) of the prepontine region. The average percentage increase in the CSF signal within the prepontine SAS, compared to pre-injection levels, is shown for all subjects. *P < 0.05, **P < 0.01, ***P < 0.001 from test of tracer enrichment. Trend plots are presented as means ± 95% confidence intervals (CIs), derived from linear mixed models, and displaying jitter plots of individual data points.

Supplementary Figure 6

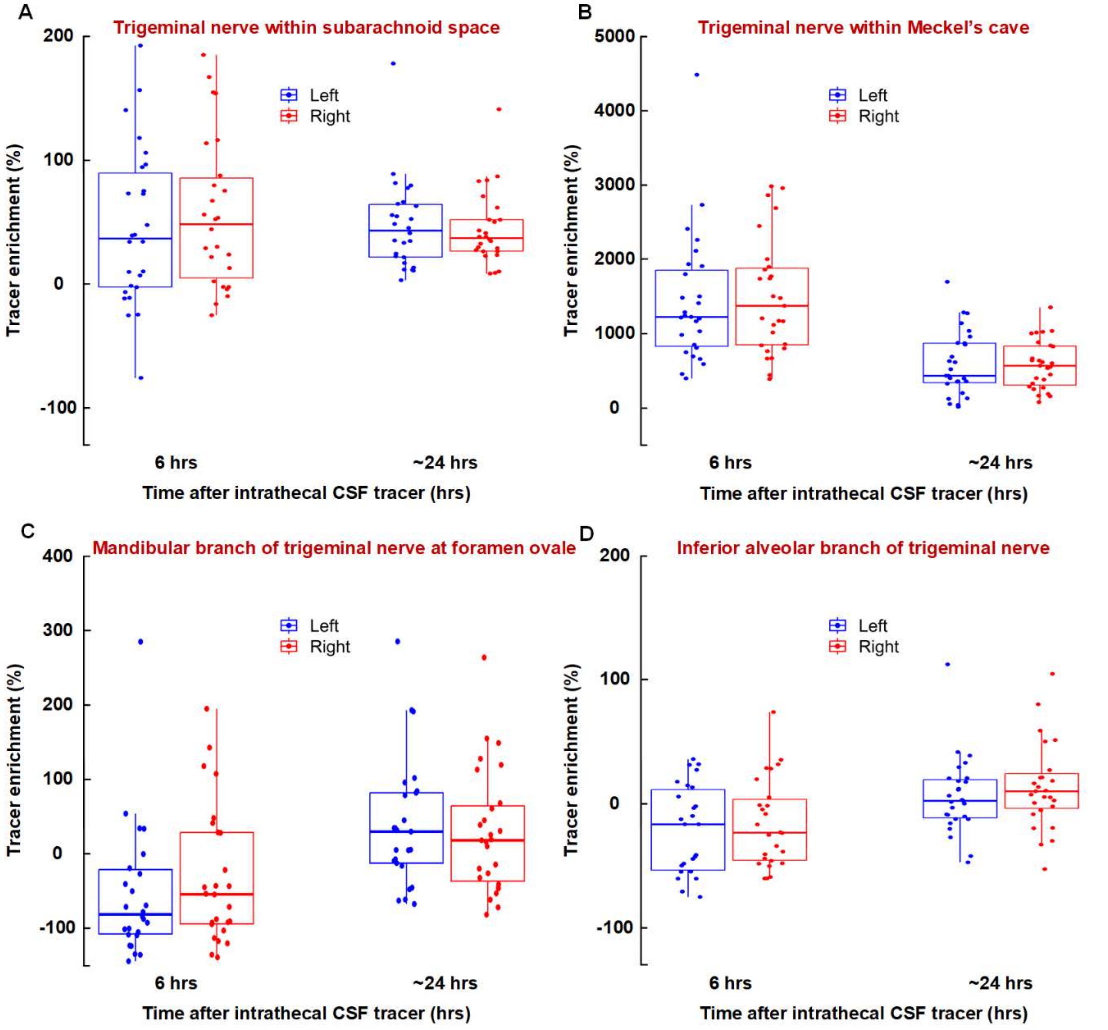

Comparison of tracer enrichment between the left and right trigeminal nerves. The percentage change in tracer enrichment, compared to pre-injection levels, at 6 and 24 hours is shown for (a) the trigeminal nerve within the prepontine subarachnoid space, (b) within Meckel's cave, (c) the mandibular branch at the foramen ovale, and (d) the inferior alveolar nerve at the mandibular bone. Data are presented as box plots with medians and the 25th and 75th percentiles, along with individual values. No significant differences in tracer enrichment were observed between the left and right sides (linear mixed model analysis).

Supplementary Figure 7

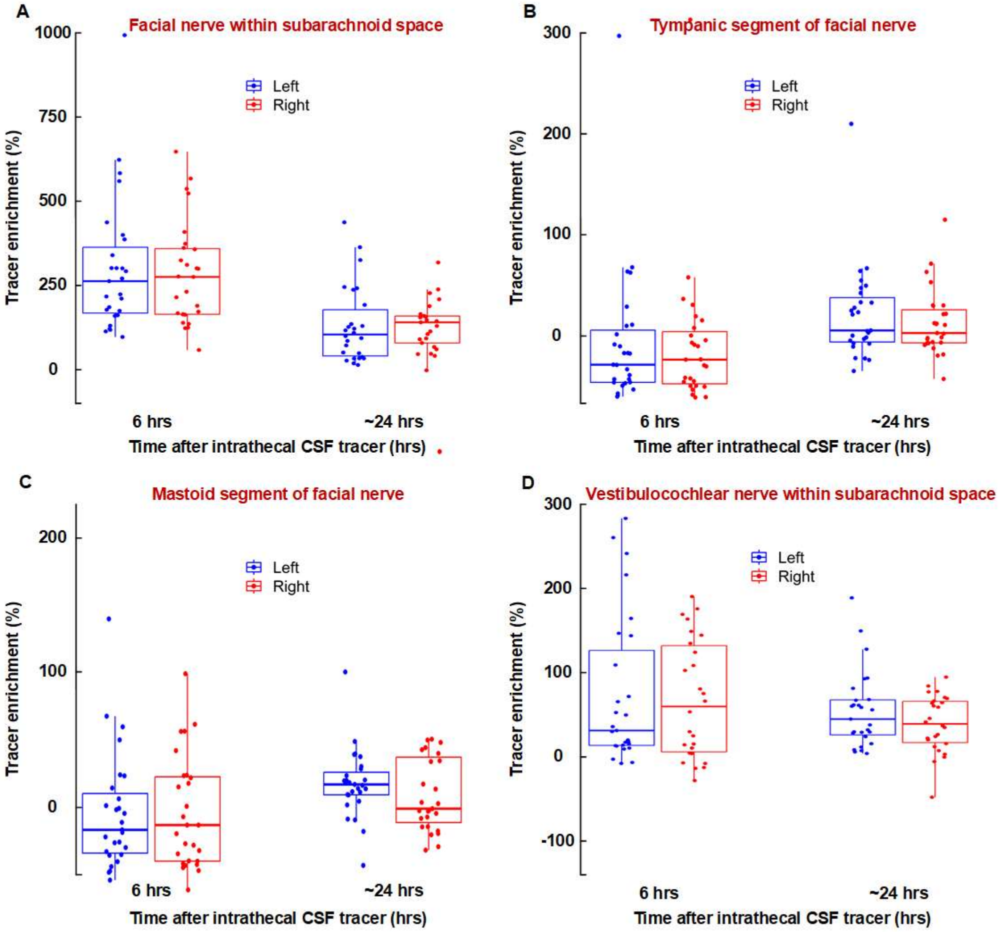

Comparison of tracer enrichment between the left and right facial and vestibulocochlear nerves. The percentage change in tracer enrichment, compared to pre-injection levels, at 6 and 24 hours is shown for (a) the facial nerve within the prepontine subarachnoid space, (b) tympanic segment of the facial nerve, (c) mastoid segment of the facial nerve, and (d) the vestibulocochlear nerve. Data are presented as box plots with medians and the 25th and 75th percentiles, along with individual values. No significant differences in tracer enrichment were observed between the left and right sides (linear mixed model analysis).
